# Ergothioneine: an underrecognised dietary micronutrient required for healthy ageing?

**DOI:** 10.1017/S0007114522003592

**Published:** 2023-01-14

**Authors:** Xiaoying Tian, James L. Thorne, J. Bernadette Moore

**Affiliations:** School of Food Science & Nutrition, University of Leeds, Leeds, LS2 9JT, UK

**Keywords:** Ergothioneine, Cardiometabolic disease, CVD, Type 2 diabetes, Non-alcoholic fatty liver disease

## Abstract

Ergothioneine is a naturally occurring amino acid and thiol antioxidant found in high amounts in mushrooms and fermented foods. Humans and animals acquire ergothioneine from the diet through the pH-dependent activity of a membrane transporter, the large solute carrier 22A member 4 (SLC22A4), expressed on the apical membrane of the small intestine. The SLC22A4 transporter also functions in the renal reabsorption of ergothioneine in the kidney, with avid absorption and retention of ergothioneine from the diet observed in both animals and humans. Ergothioneine is capable of scavenging a diverse range of reactive oxygen and nitrogen species, has metal chelation properties, and is predicted to directly regulate nuclear factor erythroid 2-related factor 2 (Nrf2) activity. Although not lethal, the genetic knockout of the SLC22A4 gene in multiple organisms increases susceptibility to oxidative stress, damage and inflammation; in agreement with a large body of preclinical data suggesting the physiological function of ergothioneine is as a cellular antioxidant and cytoprotectant agent. In humans, blood levels of ergothioneine decline after the age of 60 years, and lower levels of ergothioneine are associated with more rapid cognitive decline. Conversely, high plasma ergothioneine levels have been associated with significantly reduced cardiovascular mortality and overall mortality risks. In this horizon’s manuscript, we review evidence suggesting critical roles for dietary ergothioneine in healthy ageing and the prevention of cardiometabolic disease. We comment on some of the outstanding research questions in the field and consider the question of whether or not ergothioneine should be considered a conditionally essential micronutrient.

## Graphical abstract

**Figure:**
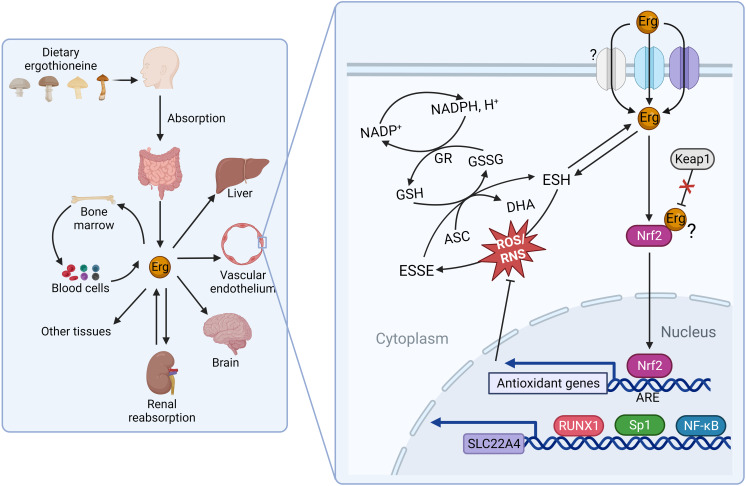

Ergothioneine is a naturally occurring, betaine amino acid found in many foods. A derivative of histidine (2-mercapto-histidine trimethylbetaine)^(1,2)^, ergothioneine, was first isolated in 1909 from the ergot fungus *Claviceps purpurea*, from which its name was derived^(3)^. Structurally ergothioneine is a tautomer, with both thione and thiol forms (Fig. 1). Unusual among the thiol antioxidants, at physiological pH, ergothioneine exists primarily in its thione form and has a very high redox potential^(4)^. These unique properties mean that ergothioneine is much more resistant to autooxidation in comparison with other thiols such as glutathione and is a very effective antioxidant and cytoprotectant, with metal chelation properties as well^(4–6)^. An increasing body of evidence suggests ergothioneine may be an important dietary nutrient for the prevention of a variety of inflammatory and cardiometabolic diseases^(6,7)^; and ergothioneine has alternately been suggested as a vitamin^(5)^, ‘longevity vitamin’^(8)^ and nutraceutical^(6)^.

**Fig. 1.**
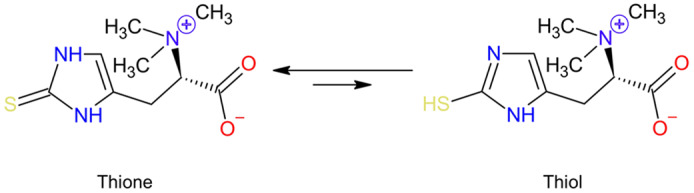
Chemical structure of ergothioneine. A histidine-derived amino acid, ergothioneine, exists as a tautomer with both thione and thiol forms. As indicated by the length of the reaction arrows, at physiological pH, the thione structure predominates.

Although ergothioneine is present in plants and animals, evidence for ergothioneine biosynthesis, to date, is limited to bacteria and fungi^(9,10)^. More recent genetic and structural approaches have built on early work done in the 1950s^(11–13)^ and demonstrated that ergothioneine biosynthesis has independently emerged at least three times in the molecular evolution of mycobacteria, anaerobic archaebacteria and cynaobacteria^(10)^. The typically low levels of ergothioneine found in plants have been presumed to be acquired through their roots from soil fungi or bacteria as part of mycorrhizal symbiosis. This has been demonstrated interestingly in an achlorophyllous plant, *Gastrodia elata*, whose lifecycle is dependent on the presence of symbiotic fungi^(14)^. However, as has long been opined, absence of evidence is not evidence of absence^(15)^. The systematic investigation of ergothioneine biosynthesis in plants using genetic approaches is ongoing^(16)^, and new data may yet challenge the presumption that plants do not synthesise ergothioneine.

Mushrooms are typically the richest source of ergothioneine in the human diet, with amounts of ergothioneine varying wildly depending on strain and growing conditions^(17)^. Differences in cultivation practices, including cultivation substrates^(18)^, and soil health and tillage methods^(19)^, likely explain much of the large variation observed in the ergothioneine contents of both mushrooms and other foods from different places of production (Tables 1 and 2). Divergent sample preparation and analytical methods likely also contribute to the variable ergothioneine concentrations reported to date. Fermented foods can also be significant sources of ergothioneine (Table 1), with the concentration of ergothioneine dependent on the different species of bacteria used in fermentation^(20)^. In addition, spirulina, the dried biomass of cyanobacteria (*Arthrospira platensis*) sold commonly as a dietary supplement, contains relatively high amounts of ergothioneine^(21)^. It is interesting to observe that ergothioneine is found in high amounts in certain plants, mushrooms and spirulina, that have been used and investigated for their medicinal properties^(14,22,23)^.

**Table 1.** Ergothioneine content in fermented foods and mushrooms

Food group	Food name	Place of production	Method of measurement	Content (mg/kg)	Reference
Fermented foods	Beer	Germany	LC-MS	0·02^wwt^	^(102)^
	Tempeh	Singapore	LC-MS	201·13^dwt^	^(17)^
	Fermented rice, bran	Japan	LC-MS	176·00^wwt^	^(103)^
	Fermented rice, white	Japan	LC-MS	59·80^wwt^	^(103)^
	Fermented rice, brown	Japan	LC-MS	85·50^wwt^	^(103)^
	Fermented shiitake with *Lactobacillus acidophilus*	Korea	HPLC	1668·40^wwt^	^(20)^
	Fermented shiitake with *Lactobacillus fermentum*	Korea	HPLC	1389·00^wwt^	^(20)^
	Fermented shiitake with *Lactobacillus plantarum*	Korea	HPLC	1278·60^wwt^	^(20)^
	Fermented shiitake with *Pediococcus pentosaceus*	Korea	HPLC	1775·20^wwt^	^(20)^
Mushrooms	Abalone	Singapore	LC-MS	324·70^dwt^	^(17)^
	Black fungus	Singapore	LC-MS	94·20^dwt^	^(17)^
	Button, crimini	USA	LC-MS	680·00^dwt^	^(104)^
	Button, crimini	USA	HPLC	550·00^dwt^	^(105)^
	Button, crimini	USA	HPLC	400·00^dwt^	^(106)^
	Button, crimini	Singapore	LC-MS	104·10^dwt^	^(17)^
	Button, crimini	Japan	HPLC	24·17^wwt^	^(107)^
	Button, portabella	USA	LC-MS	680·00^dwt^	^(104)^
	Button, portabella	USA	HPLC	450·00^dwt^	^(106)^
	Button, portabella	Singapore	LC-MS	190·90^dwt^	^(17)^
	Button, portabella	Germany	LC-MS	0·93^wwt^	^(102)^
	Button, white	Greece	UV-Vis	7100·00^dwt^	^(18)^
	Button, white	Greece	LC-MS	521·20^dwt^	^(18)^
	Button, white	USA	LC-MS	410·00^dwt^	^(104)^
	Button, white	USA	HPLC	210·00^dwt^	^(104)^
	Button, white	Singapore	LC-MS	154·40^dwt^	^(17)^
	Button, white	Japan	HPLC	78·98^wwt^	^(107)^
	Button, white	Germany	LC-MS	0·46^wwt^	^(102)^
	Chanterelle	Germany	LC-MS	0·06^wwt^	^(102)^
	Chestnut	Japan	HPLC	1290·00^dwt^	^(108)^
	Enoki	Singapore	LC-MS	346·40^dwt^	^(17)^
	Enoki	Japan	HPLC	151·17^wwt^	^(107)^
	King trumpet (*Pleurotus eringii*)	Japan	HPLC	77·07^wwt^	^(107)^
	Gargal	Japan	HPLC	2040·00^dwt^	^(108)^
	Giant leucopax	Japan	HPLC	1700·00^dwt^	^(108)^
	Grey knight *(Tricholoma sp.)*	Japan	HPLC	910·00^dwt^	^(108)^
	*Hyspatys marrcus*	Japan	HPLC	14·97^wwt^	^(107)^
	Jelly ear	Japan	HPLC	32·29^wwt^	^(107)^
	Maitake	USA	HPLC	1130·00^dwt^	^(106)^
	Maitake	Japan	HPLC	670·00^dwt^	^(108)^
Mushrooms	Maitake	Singapore	LC-MS	20·20^dwt^	^(17)^
	Maitake, black	Japan	HPLC	20·19^wwt^	^(107)^
	Maitake, white	Japan	HPLC	103·92^wwt^	^(107)^
	Nameko (butterscotch)	Japan	HPLC	460·00^dwt^	^(108)^
	Nameko (butterscotch)	Japan	HPLC	12·69^wwt^	^(107)^
	Oyster	Greece	UV-Vis	9200·00^dwt^	^(18)^
	Oyster	USA	HPLC	2590·00^dwt^	^(106)^
	Oyster	USA	LC-MS	2010·00^dwt^	^(104)^
	Oyster	Japan	HPLC	1980·00^dwt^	^(108)^
	Oyster	Greece	LC-MS	607·30^dwt^	^(18)^
	Oyster	Germany	LC-MS	118·91^wwt^	^(102)^
	Oyster, golden	Japan	LC-MS	10 000·00^wwt^	^(46)^
	Oyster, golden	Greece	UV-Vis	8300·00^dwt^	^(18)^
	Oyster, golden	Greece	LC-MS	822·10^dwt^	^(18)^
	Oyster, golden, cultivated in grape marc	Greece	UV-Vis	11 800·00^dwt^	^(18)^
	Oyster, golden, cultivated in wheat straw	Greece	UV-Vis	8300·00^dwt^	^(18)^
	Oyster, golden, cultivated in grape marc	Greece	LC-MS	637·20^dwt^	^(18)^
	Oyster, golden, cultivated in olive by-products	Greece	LC-MS	884·50^dwt^	^(18)^
	Oyster, golden, cultivated in wheat straw	Greece	LC-MS	822·10^dwt^	^(18)^
	Oyster, king	USA	LC-MS	1720·00^dwt^	^(104)^
	Oyster, king	Japan	HPLC	1410·00^dwt^	^(108)^
	Oyster, king	Singapore	LC-MS	541·70^dwt^	^(17)^
	Oyster, king	Japan	HPLC	234·85^wwt^	^(107)^
	Polypore	USA	LC-MS	1840·00^dwt^	^(104)^
	Porcini	Germany	LC-MS	528·14^wwt^	^(102)^
	Porcini	Singapore	LC-MS	1812·40^dwt^	^(17)^
	Price	Japan	HPLC	19·85^wwt^	^(107)^
	Shiitake	USA	LC-MS	2090·00^dwt^	^(104)^
	Shiitake	USA	HPLC	1980·00^dwt^	^(106)^
	Shiitake	Korea	HPLC	1260·40^wwt^	^(20)^
	Shiitake	Japan	HPLC	400·00^dwt^	^(108)^
	Shiitake	Singapore	LC-MS	353·50^dwt^	^(17)^
	Shiitake	Japan	HPLC	123·01^wwt^	^(107)^
	Shimeji	Japan	HPLC	55·52^wwt^	^(107)^
	Shimeji, buna	Singapore	LC-MS	432·60^dwt^	^(17)^
	Shimeji, buna	Japan	HPLC	84·06^wwt^	^(107)^
	Shimeji, white	Singapore	LC-MS	197·50^dwt^	^(17)^
	White fungus	Singapore	LC-MS	5·80^dwt^	^(17)^
	Willow	Singapore	LC-MS	296·80^dwt^	^(17)^
	Wood ear	Singapore	LC-MS	6·40^dwt^	^(17)^
	Wood ear	Singapore	LC-MS	6·40^dwt^	^(17)^

wwt, wet weight; dwt, dry weight; UV-Vis, ultraviolet–visible spectrometry.

**Table 2.** Ergothioneine content in foods

Food group	Food name	Place of production	Method of measurement	Content (mg/kg)	Reference
Vegetables and fruits	Asparagus	Mexico	LC-MS	163·25^dwt^	^(17)^
	Asparagus	Thailand	LC-MS	10·24^dwt^	^(17)^
	Asparagus	Malaysia	LC-MS	0·57^dwt^	^(17)^
	Asparagus, white	Singapore	LC-MS	18·20^dwt^	^(17)^
	Basil leaf	Singapore	LC-MS	4·92^dwt^	^(17)^
	Black turtle bean	Germany	LC-MS	13·49^wwt^	^(102)^
	Broccoli	Singapore	LC-MS	0·38^dwt^	^(17)^
	Broccoli	Germany	LC-MS	0·24^wwt^	^(102)^
	Celery	Germany	LC-MS	0·08^wwt^	^(102)^
	Cumin	Singapore	LC-MS	2·60^dwt^	^(17)^
	Durian	Singapore	LC-MS	1·09^dwt^	^(17)^
	Garlic	Singapore	LC-MS	34·60^dwt^	^(17)^
	Garlic	Germany	LC-MS	3·11^wwt^	^(102)^
	Ginger	Singapore	LC-MS	0·17^dwt^	^(17)^
	Ginseng root	Singapore	LC-MS	0·69^dwt^	^(17)^
	Japanese seaweed	Singapore	LC-MS	2·34^dwt^	^(17)^
	Kale	Singapore	LC-MS	0·22^dwt^	^(17)^
	Kidney beans	Singapore	LC-MS	2·09^dwt^	^(17)^
	Kiwi fruit	Singapore	LC-MS	1·99^dwt^	^(17)^
	Onion	Singapore	LC-MS	1·13^dwt^	^(17)^
	Onion	Germany	LC-MS	0·23^wwt^	^(102)^
	Parsnip	Singapore	LC-MS	2·23^dwt^	^(17)^
	Passion fruit	Singapore	LC-MS	1·22^dwt^	^(17)^
	Pepper	Singapore	LC-MS	2·57^dwt^	^(17)^
	Persimmon	Singapore	LC-MS	1·52^dwt^	^(17)^
	Pomegranate	Singapore	LC-MS	1·30^dwt^	^(17)^
	Red kidney bean	Germany	LC-MS	4·52^wwt^	^(102)^
	Spinach	Germany	LC-MS	0·11^wwt^	^(102)^
	Sweet bean	Singapore	LC-MS	1·33^dwt^	^(17)^
	Tomato	Singapore	LC-MS	0·20^dwt^	^(17)^
Grains, nuts and seeds	Almond	Singapore	LC-MS	1·87^dwt^	^(17)^
	Brazilian nut	Singapore	LC-MS	4·45^dwt^	^(17)^
	Brown rice	Germany	LC-MS	0·04^wwt^	^(102)^
	Gingko nut	Singapore	LC-MS	3·98^dwt^	^(17)^
	Macadamia nut	Singapore	LC-MS	1·65^dwt^	^(17)^
	Oat	Singapore	LC-MS	1·84^dwt^	^(17)^
	Oat bran	Germany	LC-MS	4·41^wwt^	^(102)^
	Pistachio nut	Singapore	LC-MS	1·90^dwt^	^(17)^
Grains, nuts and seeds	Pumpkin seed	Germany	LC-MS	1·49^wwt^	^(102)^
	Spelt	Germany	LC-MS	0·61^wwt^	^(102)^
	Wheat bran	Germany	LC-MS	0·84^wwt^	^(102)^
	Wheat germ	Germany	LC-MS	0·63^wwt^	^(102)^
Meats	Beef loin steak	Germany	LC-MS	1·33^wwt^	^(102)^
	Chicken breast	Germany	LC-MS	1·15^wwt^	^(102)^
	Chicken liver	Germany	LC-MS	10·78^wwt^	^(102)^
	Lamb loin fillet	Germany	LC-MS	1·20^wwt^	^(102)^
	Pork kidney	Germany	LC-MS	7·66^wwt^	^(102)^
	Pork liver	Germany	LC-MS	8·71^wwt^	^(102)^
	Pork loin fillet	Germany	LC-MS	1·68^wwt^	^(102)^
Eggs and milk	Egg yolk	Germany	LC-MS	0·68^wwt^	^(102)^
	Egg white	Germany	LC-MS	0·38^wwt^	^(102)^
	Fresh milk	Singapore	LC-MS	0·25^dwt^	^(17)^
Seafood	Trout	Germany	LC-MS	0·07^wwt^	^(102)^
Processed foods	Aspic (pork)	Germany	LC-MS	0·46^wwt^	^(102)^
	Blood sausage (pork)	Germany	LC-MS	1·08^wwt^	^(102)^
	Ham	Germany	LC-MS	1·12^wwt^	^(102)^
	Liver sausage (pork)	Germany	LC-MS	1·03^wwt^	^(102)^
	Salami (pork)	Germany	LC-MS	0·51^wwt^	^(102)^
	Soya bean curd	Singapore	LC-MS	3·71^dwt^	^(17)^
	Soya milk	Singapore	LC-MS	2·3^dwt^	^(17)^
	Whole grain brown bread	Germany	LC-MS	0·47^wwt^	^(102)^
	Whole grain wheat bread	Germany	LC-MS	0·53^wwt^	^(102)^

dwt, dry weight; wwt, wet weight.

## Absorption, transport and tissue distribution

**Fig. 2.**
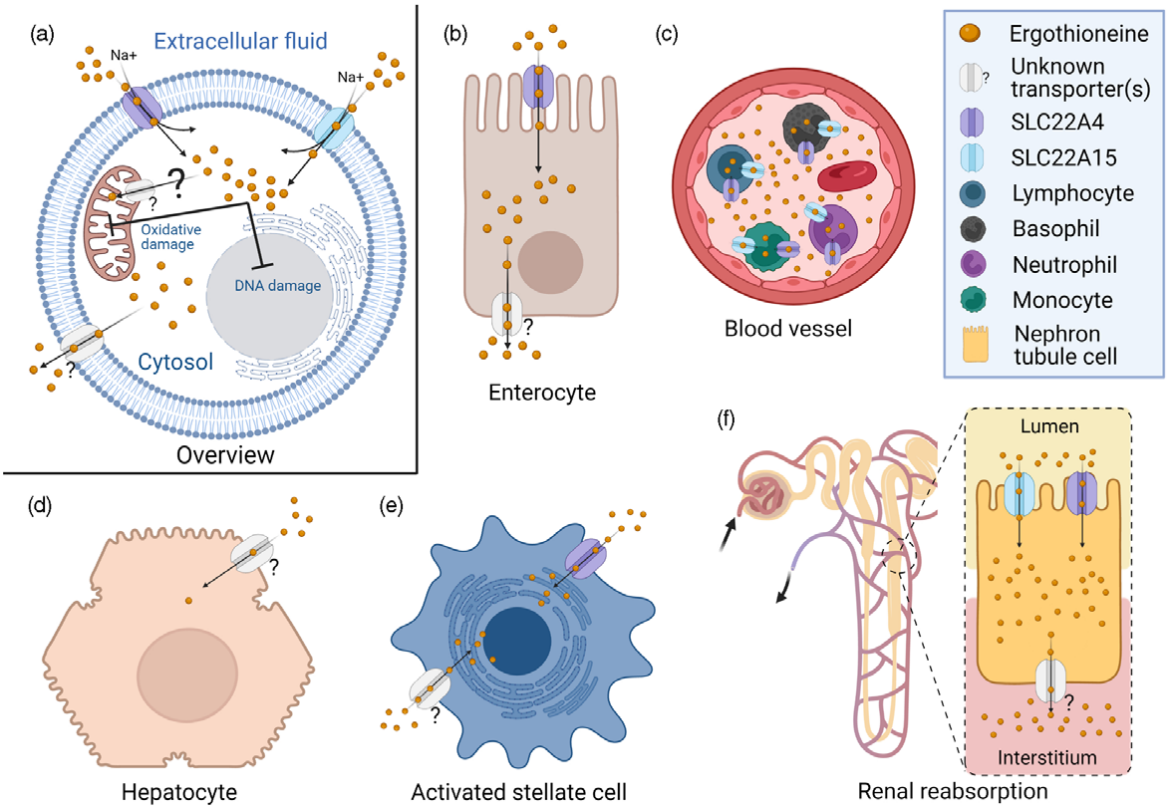
Physiological transport of ergothioneine *in vivo*. (a) Ergothioneine is transported across cell membranes by the solute carrier family 22 member’s 4 and 15 (SLC22A4 and SLC22A15) in a Na-dependent manner. Intracellularly, ergothioneine inhibits oxidative and DNA damage through multiple pathways. Whether ergothioneine is transported into the mitochondria is unknown. How ergothioneine is exported from cells is also unknown. (b) Uptake of ergothioneine from the diet into enterocytes is mediated by SLC22A4 expression on the apical membrane. The basolateral transporter is unknown. (c) Ergothioneine is rapidly cleared from plasma. SLC22A4 is highly expressed in granulocytes, monocytes and nucleated erythroid precursors, but not mature erythrocytes. (d) Although ergothioneine accumulates in mouse liver, SLC22A4 is not expressed on hepatocytes. In humans, while SLC22A4 is highly expressed in fetal liver, it is only detected in very low amounts in adult liver. (e) Some data suggest that SLC22A4 is expressed in liver non-parenchymal cells^(35)^ and is upregulated in activated hepatic stellate cells in mice^(46)^. (f) Ergothioneine is avidly retained through renal reabsorption mediated by SLC22A4 expression at the apical membrane of proximal tubular cells in the kidney. The basolateral transporter is unknown.

The seminal identification of a mammalian membrane transporter for ergothioneine by Grundemann and colleagues in 2005^(24)^ provided a mechanism by which ergothioneine is acquired from the diet, and further evidence for an essential role for ergothioneine *in vivo*. Genetic knockout of solute carrier 22A member 4 (SLC22A4), part of the large solute carrier 22A (SLC22A) family, in both mice^(25)^ and zebrafish^(26)^, resulted in dramatic reduction of ergothioneine to undetectable concentrations in many tissues (e.g. liver ergothioneine was 121 ± 25 in wild-type mice *v*. <2·13 μg/g in SLC22A4 knockout mice)^(25)^, and increased susceptibility to oxidative stress and inflammation, although organisms remained viable. Nomenclature can be challenging as the SLC22A family is comprised of organic cation (OCT), organic anion (OAT) and ‘novel organic cation’ (i.e. cation and zwitterion; OCTN) transporters. SLC22A4 was originally termed OCTN1^(27)^ and is found referred to as both OCTN1 and the ergothioneine transporter in the literature. With the recent de-orphaning of SLC22A15 as a second ergothioneine transporter expressed highly in the brain^(28)^, and likelihood of further ergothioneine transporters yet to be characterised (see Fig. 2 for overview and outstanding questions related to ergothioneine membrane transport), we will use the HUGO Gene Nomenclature Committee (HGNC)-approved human gene nomenclature here.

Differentially expressed between tissues, SLC22A4 is notably highly expressed both on the apical membrane of the small intestine where it functions in a pH-dependent fashion to take ergothioneine up from the diet^(29)^ and on the apical membrane of proximal tubular cells in the kidney where it functions in renal reabsorption^(30)^. Although, like other SLC22 family members, SLC22A4 is capable of transporting multiple substrates, the transport efficiencies for other confirmed substrates are orders of magnitude lower than that of ergothioneine, and increasing evidence from independent laboratories demonstrates SLC22A4 is highly specific for ergothioneine^(28,31,32)^. In humans, SLC22A4 is also very highly expressed in nucleated erythroid precursor cells, bone marrow and fetal (but not adult) liver^(24,27)^; and experimental data are consistent with a role for ergothioneine in erythroid proliferation and differentiation^(33)^. In addition, SLC22A4 is expressed highly in circulating neutrophils and monocytes, suggestive of a requirement for ergothioneine’s antioxidant function in these cells, which are predisposed to oxidative stress^(34)^.

Several lines of evidence suggest that there are other ergothioneine transporters yet to be characterised^(34)^. Perhaps most notably, a basolateral transporter responsible for the efflux of ergothioneine in polarised cells such as enterocytes and proximal tubules cells of the liver has yet to be identified. Secondly, plasma concentrations of tritiated ergothioneine were reported to increase in response to oral administration of ergothioneine in SLC22A4 knockout mice, albeit not as highly as in wild-type mice^(35)^. Although transport by SLC22A15 cannot be ruled out as an explanation, it is (at least in humans) expressed at very low levels in the small intestine^(28)^ and has a much lower transport efficiency for ergothioneine in comparison with SLC22A4, so unlikely to explain the kinetics reported by Sugiura and colleagues^(35)^. The authors suggest the plasma increase was because the knockout mice were lacking the efficient liver uptake observed in the wild-type mice. However, it is not clear how ergothioneine could have crossed the enterocyte in the absence of SLC22A4 or another transporter. Lastly, the question of whether or not there is a mitochondrial (or other subcellular location)-specific transporter for ergothioneine is unresolved^(36)^. While early reports of mitochondrial localisation of SLC22A4 are disputed^(34,36)^, recent data from a rat model of pre-eclampsia showed ergothioneine supplementation decreased mitochondria-specific H_2_O_2_ *in vivo* ^(37)^, and the question of mitochondrial targeting of ergothioneine remains plausible based on previous *in vitro* data^(5)^.

**Fig. 3.**
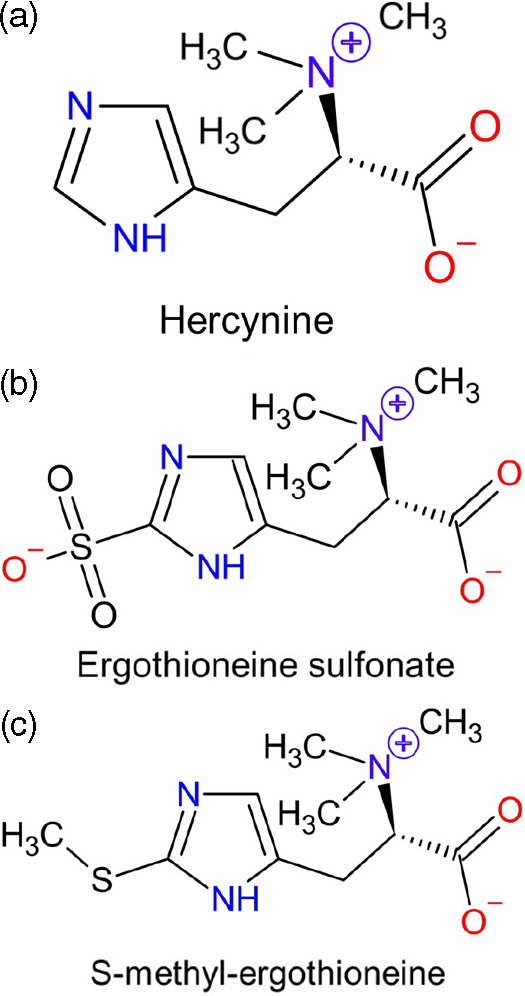
The putative metabolites of ergothioneine. (a) Hercynine. (b) Ergothioneine sulphonate. C. S-methyl-ergothioneine.

In both animals and humans, avid absorption and retention of ergothioneine have been observed^(38,39)^. Regulatory safety approval for ergothioneine supplementation in humans has only occurred quite recently (2016 in Europe^(40)^ and 2018 in the USA^(41)^), and to date only one study has examined ergothioneine supplementation in humans^(39)^. In this pharmacokinetic study, forty-five healthy humans received placebo, 5, or 25 mg encapsulated ergothioneine/d for 7 d and were followed up for an additional 4 weeks. The data show that ergothioneine was rapidly absorbed and largely retained by the body, with large increases in plasma ergothioneine levels and only minimal increases (<4 %) in urinary excretion observed^(39)^. In mice, daily oral administration of a high dose of ergothioneine (70 mg/kg/d) for 28 d showed that while ergothioneine primarily accumulated in liver and whole blood, levels also increased in multiple tissues including kidney and brain^(38)^. In both studies^(38,39)^, the putative metabolites of ergothioneine (Fig. 3; chemistry reviewed in detail by Servillo^(42)^), the oxidative degradation products hercynine (desulfurated ergothioneine) and ergothioneine sulfonate, as well as its methylated form, S-methyl-ergothioneine, were measured. In humans, hercynine and S-methyl-ergothioneine levels correlated with ergothioneine levels in blood, but ergothioneine sulfonate levels were at the lower limits of detection^(39)^.

Interestingly, while SLC22A4 is highly expressed in rat^(43)^ and mouse liver^(44)^, in humans, although highly expressed in fetal liver^(27)^, it is typically barely detectable in adult liver^(7,45)^. In the human pharmacokinetic study from Cheah and colleagues^(39)^, while plasma levels of ergothioneine decreased when supplementation was withdrawn, levels in whole blood continued to increase in a dose–response fashion reaching maximal levels 3 weeks after withdrawal of supplement, which were sustained at 4 weeks follow-up. Whether or not bone marrow, where both SLC22A4 and SLC22A15 are highly expressed^(28)^, or another extra-hepatic tissue is the site of ergothioneine retention in humans remains an open question. Likewise in mice, questions also remain about what cell type in liver SLC22A4 are expressed in and where ergothioneine accumulates. SLC22A4 has been reported to be expressed in non-parenchymal cells^(35)^, and to be upregulated in activated hepatic stellate cells^(46)^. However, these cell types are far fewer in number in the liver than hepatocytes, which represent 70–80 % of liver and are responsible for first-pass metabolism; a fact difficult to reconcile with the efficient hepatic uptake reported in mice administered intravenous ergothioneine^(35,47)^.

## Biological roles

Extensive *in vitro* data (reviewed in detail by Cheah and Halliwell^(4)^ and Borodina and colleagues^(6)^) strongly suggests ergothioneine functions as an antioxidant and acts intracellularly as a cytoprotective agent. Ergothioneine reacts almost instantaneously with hydroxyl radicals *in vitro* ^(48)^ and also scavenges a diverse range of additional reactive oxygen and nitrogen species^(4,6)^. Ergothioneine deactivates singlet-oxygen species at higher rates than other thiols^(49)^, and it has been hypothesised that the primary function of ergothioneine may be to prevent damage at intracellular sites of high singlet-oxygen generation^(26)^. Redox repair of the oxidised forms (ergothioneine disulfide and 5-oxo-ergothioneine) of ergothioneine can be rapidly provided by ascorbate^(48)^ or can be achieved enzymatically by either glutathione reductase in presence of glutathione, or the selenoenzyme thioredoxin reductase^(50)^.

While the historical study of dietary antioxidants bears cautionary lessons^(51)^, in support of a critical antioxidant role for ergothioneine *in vivo*, the genetic knockout of SLC22A4 in Caenorhabditis elegans increased oxidative damage and reduced lifespan^(52)^. In addition, knockout of SLC22A4 in zebrafish^(26)^ and mice^(25)^ also resulted in increased susceptibility to oxidative stress and inflammation for both organisms. Notably, under basal conditions, high expression of SLC22A4 is typically observed in cells with routinely high amounts of oxidative stress such as granulocytes, bone marrow cells, intestinal and ocular tissues^(7)^. However, accumulation of ergothioneine and increased expression of SLC22A4 in other (injured) tissues have been observed in animal models of liver fibrosis^(46)^, fatty liver disease^(53)^ and chronic kidney disease disease^(54)^; as well as in humans with Crohn’s disease^(45)^. Prompting the hypothesis that the accumulation of ergothioneine is an adaptive mechanism to minimise oxidative damage^(55)^.

Initial reports that a gain-of-function polymorphism (L503F variant, rs1050152) in *SLC22A4* that increased transport efficiency of ergothioneine was associated with increased risk of Crohn’s disease^(56)^ were subsequently shown to have been confounded by the *SLC22A4* gene being in linkage disequilibrium with the interferon regulatory factor 1 (*IRF1*) gene, which was the locus conferring Crohn’s disease susceptibility^(57)^. Similarly, an initial report that an intronic SNP (rs2268277) in *SLC22A4* was associated with rheumatoid arthritis^(58)^ did not replicate in independent populations^(59)^. In this case, the rs2268277 SNP is located at a runt-related transcription factor 1 (RUNX1) binding site located in intron 6 of the *SLC22A4* gene^(58)^. Reporter gene assay data suggests RUNX1 has a stronger suppressive effect on the minor rs2268277 allele^(58)^.

In addition, multiple other SNP have been found in the *SLC22A4* gene, with functional characterisation of eight non-synonymous SNP in Chinese and Indian populations of Singapore finding four of the variants had reduced transporter activity^(60)^. A large difference in basal concentrations of ergothioneine in whole blood was observed in a pharmacokinetic study by Cheah and colleagues^(61)^, who also noted that participants with the highest basal levels of ergothioneine also appeared to take up more of the supplemented ergothioneine. However, whether or not polymorphisms in SLC22A4 affect ergothioneine tissue concentrations and/or are linked to disease risk in some individuals remains an open question that should be examined in much larger genetic cohorts (e.g. the Biobank cohort with *n* 500 000^(62)^).

Along with RUNX1, transcription of the SLC22A4 gene has also been shown to be regulated by the NF-*κ*B transcription factor, and the inflammatory cytokines IL-1 *β* and TNF-*α* ^(63)^, data suggestive of ergothioneine accumulation being part of an orchestrated cellular immune defence. Moreover, multiple *in vitro* ^(64,65)^ and *in vivo* ^(66,67)^ studies suggest that ergothioneine activates the transcription factor nuclear factor erythroid 2-related factor 2 (Nrf2), which triggers the cellular antioxidant defence system. Recent molecular docking and dynamic simulation data predict ergothioneine binds Nrf2 directly, preventing Nrf2 degradation^(67)^. These data underscore the pleiotropic cytoprotective effects of ergothioneine beyond its redox activity.

## Healthy ageing and the prevention of cardiometabolic disease

In addition to the discussed antioxidant activities, ergothioneine has been demonstrated to chelate divalent cations^(68)^ and protect against gamma^(69)^ and UV^(70)^ radiation with dermato-protective effects observed in human dermal fibroblasts and keratinocytes^(64,70,71)^, prompting the use of ergothioneine in some skincare formulas. Beyond cosmetic concerns, the potential anti-ageing effects of ergothioneine in the brain have also been of significant research interest, with *in vivo* data from animal models showing that ergothioneine protects neurons from damage by cisplatin^(72)^, β-amyloid^(73)^, and age-related learning and memory deficits^(74)^. In humans, blood levels of ergothioneine start declining linearly with the age after 60 years^(75)^. A number of small human case–control studies have found lower ergothioneine levels in older adults with mild cognitive impairment^(75)^, dementia^(76)^ and Parkinson’s disease^(77)^, in comparison with age-matched healthy individuals. In support of these data, in a prospective elderly cohort in Singapore (*n* 470, mean age 73), lower baseline ergothioneine levels were associated with poorer baseline cognitive performance and faster rates of decline in function in multiple cognitive domains over 5 years of follow-up^(78)^. Whether or not ergothioneine supplementation (25 mg given three times a week for 52 weeks) may be beneficial in delaying or reversing cognitive decline is the subject of an ongoing clinical trial in elderly individuals with mild cognitive impairment^(79)^.

Separately, in a larger, longer-term prospective Swedish cohort (*n* 3236 participants with median follow-up of 21·4 years), higher plasma levels of ergothioneine were associated with significantly lower risk of coronary disease, cardiovascular mortality and overall mortality (hazard ratios per 1 sd increment of ergothioneine were 0·85, 0·79 and 0·86, respectively)^(80)^. These data re-enforce preclinical studies that suggest the antioxidant and anti-inflammatory activities of ergothioneine interfere with atherogenesis and protect vascular and microvascular endothelial cells from oxidative stress and hyperglycaemia^(81)^. Interestingly, in a meta-analysis of prospective cohort studies (*n* 601 893 participants) mushroom consumption was associated with lower risk of all-cause mortality (pooled risk ratio: 0·94; 95% CI: 0·91, 0·98)^(82)^.

Increased reactive oxygen species are a hallmark feature of the pathogenesis of multiple cardiometabolic diseases, including atherosclerosis, diabetes, fatty liver and CVD. Early work in perfused rat heart preparations suggested that ergothioneine protected against ischemia-induced (oxidative) myocardial damage^(83)^, which was supported by later studies showing ergothioneine protects against reperfusion injury in rat liver^(84)^ and intestinal^(85)^ ischemic reperfusion models. Although an older study of perfused rabbit hearts showed no reduction in the damage caused by reperfusion following ischaemic insult^(86)^, more recent work in diabetic rats showed that 6 weeks of ergothioneine supplementation resulted in decreased biomarkers of cardiac injury, lipid peroxidation and inflammation^(87)^.

Ergothioneine is taken up by endothelial cells via SLC22A4^(88)^, where it has been demonstrated to limit reactive oxygen species production and damage from a variety of insults, including hyperglycaemia, through multiple mechanisms^(88,89)^. Decreased expression of adhesion molecules, such as endothelial-leucocyte adhesion molecule-1 (E-selectin), intercellular adhesion molecule-1 and vascular cell adhesion molecule-1, has been observed in human aortic endothelial cells cultured with ergothioneine^(90)^. Moreover, reduced adhesion of monocytes, a key initiation step in atherosclerosis, to the human aortic endothelial cells was observed^(90)^. The cytoprotective (reduced reactive oxygen species and reduced cell senescence) effects of ergothioneine on endothelial cells exposed to hyperglycaemic conditions have been shown to be dependent on sirtuin 1 and sirtuin 6 activities and their cellular targets^(89)^, which notably include NF-*κ*B, the aforementioned transcriptional regulator of SLC22A4^(63)^.

Beyond vascular endothelial cells, supplementing the drinking water of diabetic rats with ergothioneine for 7 weeks improved multiple markers of liver injury^(91)^. Specifically, marked reductions in liver weights and TAG contents were observed, alongside reductions in liver biomarkers of lipid peroxidation (malondialdehyde content) and inflammation (TNF-*α* and transforming growth factor beta, TGF-*β*1). In addition, the ergothioneine-supplemented rats had much lower serum concentrations of the liver enzymes alanine aminotransferase, aspartate aminotransferase and alkaline phosphatase. Ergothioneine supplementation attenuated diabetes-related alterations in sirtuin-1 and NF-*κ*B mRNA expression in the liver, as well as the sterol regulatory element-binding transcription factor 1 and fatty acid synthase, the rate-limiting enzyme in fatty acid synthesis^(91)^. Separate work from the same investigators showed benefit from ergothioneine taken alone or in combination with metformin for improving markers of kidney injury (hypertrophy, serum creatinine, blood urea nitrogen and urine albumin and protein)^(67)^. Notably, the reduction of lipid peroxidation with ergothioneine supplementation has been reported in multiple injury models^(67,84,85,91)^ and is of relevance to non-alcoholic fatty liver disease (NAFLD) pathogenesis.

Closely associated with cardiometabolic disease, NAFLD is an independent risk factor CVD^(92)^, and a prevalent co-morbidity of type 2 diabetes^(93)^, with recent estimates suggesting 47–64 % of individuals with type 2 diabetes have NAFLD globally^(94)^. While ergothioneine has been demonstrated to be protective in a number of other *in vivo* models of liver injury^(46,66,95,96)^, including the aforementioned model of diabetic liver damage^(91)^; to date, only one study has examined SLC22A4 and ergothioneine in a preclinical model of NAFLD^(53)^. In this study, guinea pigs fed diets with either moderate or high levels of fat and cholesterol diets, increased liver expression of SLC22A4 mRNA, and had increased liver ergothioneine contents after 2 and 6 months of diet. This was hypothesised by the authors to be protective, as oxidative biomarkers (liver F2-isoprostane and protein carbonyl contents) were not different between dietary groups^(53)^.

In short, sufficient preclinical and epidemiological data exist to hypothesise that ergothioneine may play an important role in the prevention of cardiometabolic disease (CVD, type 2 diabetes and NAFLD) and promotion of healthy ageing. Recent regulatory safety approval for ergothioneine supplementation in humans has facilitated the requisite human intervention trials required to test such hypotheses. In addition to the aforementioned trial investigating the effects of ergothioneine supplementation on cognitive decline^(79)^, we have recently published our ErgMS study protocol^(97)^, which aims to investigate the effects of ergothioneine supplementation in middle-aged adults with metabolic syndrome. Designed as a three-arm randomised, double-blind, placebo-controlled intervention trial, the ErgMS study will supplement participants with placebo, 5 or 30 mg/d ergothioneine for 12 weeks, taking measurements of metabolic syndrome risk factors, serum markers of oxidative stress (lipid peroxidation), inflammation, blood platelet function and liver function at baseline, and after 6 weeks and 12 weeks of supplementation^(97)^.

## Future horizons

With many questions about ergothioneine function in humans outstanding, it is a fascinating time to be considering the horizons for ergothioneine-related research. As highlighted, given differences between humans and experimental models, there remain multiple unresolved questions about the molecular biology of ergothioneine transport. Membrane transporters, including the solute carrier superfamily, are notoriously difficult to study experimentally, and it seems probable that we have more to learn about how hydrophilic ergothioneine traverses polarised cells, as well as subcellular organelles. Related to transporter biology, whether the liver acts as the site of ergothioneine retention in humans, as in mice, is uncertain since SLC22A4 mRNA is barely detectable in adult human liver. Indeed, knowledge of rate and patterns of induction of SLC22A4 expression in human tissues under different (e.g. ageing and disease) circumstances is limited. Whether or not polymorphisms in SLC22A4 adversely affect an individual’s ergothioneine status and/or are linked to disease risk is also unknown. In addition, experimental confirmation that ergothioneine directly interacts with Nrf2 in a variety of tissues would further underscore the centrality of ergothioneine in cellular antioxidant defence.

Ergothioneine has alternately been suggested as a vitamin^(5)^, ‘longevity vitamin’^(8)^ and a nutraceutical^(6)^. In weighing up the relative semantics of these terms, it is worth revisiting seminal discussions of essential *v*. Conditionally essential nutrients. Classic feeding studies established nutrients as essential if, when removed from a purified diet, growth failure, failure to maintain nitrogen balance or illness occurred. Deficient nutrients were then fed back incrementally to establish minimum requirements. By the early 1980s, on the basis of such studies, thirteen vitamins, twelve minerals, nine amino acids, one fatty acid and three electrolytes were considered essential for healthy humans^(98)^. In parallel, conditionally essential nutrients were defined as those that may be synthesised in adequate amounts endogenously but could become rate-limiting under clinical conditions of stress – whether from anabolic processes (e.g. growth, pregnancy and lactation), infection or trauma^(99)^. Critically, conditionally essential nutrients, when supplied exogenously, correct a clinically relevant outcome measure^(99,100)^

In the context of clinical nutrition, Grimble in 1993 proposed five criteria to define conditionally essential nutrients in humans, whereby their deficiency results in either: failure to maintain growth or nitrogen balance, organ dysfunction, delayed recovery, metabolic or clinical abnormalities^(99)^. Metabolic and clinical abnormalities encompass obesity and cardiometabolic disease, and it is interesting to note that in CVD therapy, positive data are accumulating for therapeutic benefit from a number of potential conditionally essential nutrients, including amino acids (e.g. arginine, carnitine and coenzyme Q10, among others)^(101)^. In contrast, currently, there are no data in relation to ergothioneine intervention in humans. Such data will be critical for determining whether ergothioneine might be a conditionally essential micronutrient required for healthy ageing.

### Conclusions

Ergothioneine is a dietary antioxidant acquired from food and retained with tissue specificity in the body through the activity of the SLC22A4 membrane transporter. Genetic knockout of SLC22A4 in multiple organisms has been shown to increase organism susceptibility to oxidative stress, damage and inflammation. High plasma ergothioneine levels have been associated with significantly reduced cardiovascular mortality, and overall mortality, risks in humans. Conversely, low levels of ergothioneine have been associated with poorer cognitive performance and faster rates of cognitive decline in elderly individuals. Although a confluence of data suggests that ergothioneine acts as a powerful, pleiotropic cytoprotectant agent, and supplemental ergothioneine is already marketed direct to consumers for its anti-ageing and anti-inflammatory effects, controlled human intervention trials are just beginning to directly investigate the effects of ergothioneine supplementation in humans. Such data are required to assess whether ergothioneine is a dietary micronutrient required for healthy ageing and the prevention of cardiometabolic disease in humans.
